# Functional characterization of a tyrosinase gene from the oomycete *Saprolegnia parasitica* by RNAi silencing

**DOI:** 10.1016/j.funbio.2014.01.011

**Published:** 2014-07

**Authors:** Marcia Saraiva, Irene de Bruijn, Laura Grenville-Briggs, Debbie McLaggan, Ariane Willems, Vincent Bulone, Pieter van West

**Affiliations:** a Aberdeen Oomycete Laboratory, University of Aberdeen, Foresterhill, AB25 2ZD Aberdeen, UK; b Division of Glycoscience, School of Biotechnology, KTH – Royal Institute of Technology, AlbaNova University Center, SE-106 91 Stockholm, Sweden

**Keywords:** Melanin, Cell wall, Gene expression, Transient transformation, Oomycete

## Abstract

Here we describe the first application of transient gene silencing in *Saprolegnia parasitica,* a pathogenic oomycete that infects a wide range of fish, amphibians, and crustaceans. A gene encoding a putative tyrosinase from *S. parasitica, SpTyr,* was selected to investigate the suitability of RNA-interference (RNAi) to functionally characterize genes of this economically important pathogen. Tyrosinase is a mono-oxygenase enzyme that catalyses the *O*-hydroxylation of monophenols and subsequent oxidation of *O*-diphenols to quinines. These enzymes are widely distributed in nature, and are involved in the melanin biosynthesis. Gene silencing was obtained by delivering in vitro synthesized *SpTyr* dsRNA into protoplasts. Expression analysis, tyrosinase activity measurements, and melanin content analysis confirmed silencing in individual lines. Silencing of *SpTyr* resulted in a decrease of tyrosinase activity between 38 % and 60 %, dependent on the level of *SpTyr*-expression achieved. The *SpTyr*-silenced lines displayed less pigmentation in developing sporangia and occasionally an altered morphology. Moreover, developing sporangia from individual silenced lines possessed a less electron dense cell wall when compared to control lines, treated with GFP-dsRNA. In conclusion, the tyrosinase gene of *S. parasitica* is required for melanin formation and transient gene silencing can be used to functionally characterize genes in *S. parasitica*.

## Highlights

• Successful transient gene silencing in *Saprolegnia parasitica* through RNAi.
• Silencing a tyrosinase in *S. parasitica* results in reduced melanin formation.
• Silencing a tyrosinase in *S. parasitica* results in aberrant sporangia formation.

## Introduction

*Saprolegnia parasitica* is a fish pathogenic oomycete (water mould), belonging to the Saprolegniales order and known to infect a wide range of fish, amphibians, and crustaceans relevant to aquaculture and to aquatic ecosystems (van West et al. 2008). It causes Saprolegniosis, a disease characterised by visible white or grey patches of filamentous mycelium on the body or fins of freshwater fish (van West, 2006, Schornack et al., 2009).

**Fig 1:**
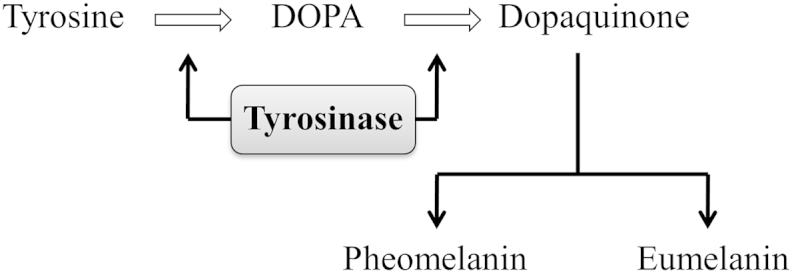
**Schematic representation of the melanin biosynthesis pathway showing the involvement of the tyrosinase enzyme.** Melanin is produced from the non-essential amino acid tyrosine by several biochemical conversions. Note that tyrosinase enzyme is involved in two steps, eventually forming dopaquinone which can be converted into either black-brown eumelanin or red-yellow pheomelanin through different via.

Within the group of oomycetes, gene transformation technology has been developed but its efficiency is, at present, limited to a restricted number of oomycete species (Judelson et al., 1991, Whisson et al., 2005, Judelson and Ah-Fong, 2009). Attempts to successfully establish transformation protocols for some oomycetes have had, in some cases, little or no success. An alternative way to functionally characterise genes, which is independent of a stable transformation protocol, can be the use of RNA-interference (RNAi). This technique was successfully developed for transient gene silencing of several genes in *Phytophthora infestans* (Whisson et al., 2005, Grenville-Briggs et al., 2008, Walker et al., 2008, Whisson et al., 2008). In the current study we performed detailed experiments to investigate whether the RNAi-technique can also be employed to silence genes in *S. parasitica*. To select a suitable gene we searched the genome of *S. parasitica* of strain CBS223.65 (Jiang *et al.* 2013) and found a gene (SPRG_01728) that encodes for a putative tyrosinase (*SpTyr*), which is highly expressed in sporulating mycelium. Tyrosinases are mono-oxygenases (monophenol, *O*-diphenol: oxygen oxidoreductases, EC 1.14.18.1) and bifunctional enzymes, catalysing the *O*-hydroxylation of monophenols and subsequent oxidation of *O*-diphenols to quinones (Fig 1). In the catalytic cycle, molecular oxygen is used as an electron acceptor leading to subsequent reduction of oxygen to water (Westerholm-Parvinen *et al.* 2007). These enzymes are crucial not only in the biosynthesis of pigments such as melanin but also in the biosynthesis of other phenol polymers such as lignin, flavonoids, and tannins (Obata *et al.* 2004). Melanins are negatively charged and high molecular hydrophobic compounds. As a result of oxidative polymerisation of phenolic compounds melanin is formed. They are insoluble in both aqueous and organic solvents and consequently difficult to study biochemically and biophysically (Casadevall *et al.* 2000). Many of the dark pigments found in nature are considered melanins (Wheeler & Bell 1988) and in microorganisms, melanin can be found in the extracellular or intracellular matrix, melanised cells of the fungal human pathogen *Cryptococcus neoformans* were shown to possess a thick layer of melanin in the cell wall (Wang *et al.* 1995). Carzaniga *et al.* (2002) demonstrated that melanin in the opportunistic plant pathogen *Alternaria alternata*, is located in the septa and outer walls of conidia (Carzaniga *et al.* 2002). In other plant pathogenic fungi like *Colletotrichum lagenarium* and *Verticillium dahliae* melanin has been found in layers within the cell wall and deposited as granules at the surface of the cell wall (Nosanchuk & Casadevall 2003).

The production of melanin has also been associated with virulence in several different microorganisms such as the pathogenic fungi *C. neoformans* and *Paracoccidioides brasiliensis* and the bacterial pathogen *Pseudomonas aeruginosa* (Nosanchuk & Casadevall 2003). Also, strains that do not produce melanin are unable to form functional appressoria and seem to lose their pathogenicity (Forrest, 1990, Takano et al., 1997, Irie et al., 2003). Melanin can also act as a protective agent against environment insults and it can bind to diverse drugs and chemicals and maintain cellular integrity (Hill 1992). Melanins have a great affinity towards metal particles and react readily with free radicals protecting the organism against oxidants such as hypochlorite and permanganate (Jacobson et al., 1994, Nyhus et al., 1997) but also against the oxidative burst of activated host effector cells (Nosanchuk & Casadevall 2003). Moreover they are less susceptible to microbicidal peptides and defensins produced by phagocytic cells. The suggested mechanism of action is the absorption of the microbicidal peptide by melanin in such a way that it cannot reach its target (Nosanchuk & Casadevall 2003).

Currently it is unclear whether oomycete tyrosinases are involved in melanin production, however it has been proposed that melanin is formed and is involved in the formation of reproductive organs and spores, in virulence, and in protection after physical damage (Lerch 1983). Indeed, during microscopic analysis of sporangial development we noticed also that the sporangia from *S*. *parasitica* are slightly darker than the mycelia. Therefore we decided to investigate whether the tyrosinase gene is involved in melanin production by silencing the gene via RNAi.

## Material and methods

### Culture maintenance

The strain of *Saprolegnia parasitica* CBS 223.65 was maintained on 4 % (w/v) potato dextrose agar (Oxoid) at 18 °C.

### Protoplast production

Mycelium from *Saprolegnia parasitica* strain CBS 223.65 was grown in Pea Broth (125 g of boiled and filtered peas per Litre) for 2 d at 24 °C, washed with sterile distilled water and collected in a 50 ml polypropylene tube (Greiner). For each 1 ml of mycelium a 3 ml solution of 10 mg ml^−1^ Cellulase (Sigma) and 5 mg ml^−1^ of Glucanase (Novozyme) diluted in 0.5 M sorbitol was prepared and added to the corresponding mycelium. The mixture was placed on a shaking platform for 90 min at room temperature (RT) to allow enzymatic cell wall degradation. The resulting protoplasts were filtrated using a 70 μm cell strainer (Fisherbrand) and washed three times with 5 ml sorbitol (0.5 M) by centrifugation (1200 g, 4 °C, 5 min) eliminating enzyme residues. The protoplasts were finally resuspended in 5 ml sorbitol (0.5 M) and two samples were counted two times each using a haemocytometer. To test protoplast regeneration ability ∼1000 protoplasts were inoculated into 20 ml Pea Broth and incubated at 18 °C for 24 h.

### RNA extraction and dsRNA production

Mycelium from *Saprolegnia parasitica* strain CBS 223.65 was grown in Pea Broth for 4 d at 24 °C, washed with sterile distilled water and left in 10 ml of sterile 1:1 tap:tank water to induce sporulation; the sporulating mycelium was then collected in a 2 ml tube with 50 μl glass beads, acid washed, and immediately frozen in liquid nitrogen. The RNA extraction was performed using the RNeasy mini Kit (Qiagen) according to manufacturer's protocol for fungi and plants with some modifications. Briefly, 600 μl of lysis buffer (RLT) buffer was added to each sample which was frozen in liquid nitrogen, mycelium was disrupted using a fast prep machine (four times, 6 ms for 45 s), samples were maintained on ice. Afterwards the samples were treated as described in the manufacture's protocol. To exclude DNA contamination the samples were subjected to a DNase treatment using the Turbo DNase kit (Ambion) according to manufacturer's protocol. The quantity and purity of the RNA was determined using the NanoDrop and the quality verified by running 1 μg of RNA in 1 % (w/v) agarose gel. Subsequent cDNA was produced using the First Strand cDNA synthesis Kit (Fermentas) according to manufacturer's protocol.

A PCR using *Tyr*-T7 primers (5′-GTAATACGACTCACTATAGGGAGCAGCTGATGTTGTAGAGC-3′ and 5′- GTAATACGACTCACTATAGGGGATCCCGTACTGGGACTACT-3′) was carried out using the cDNA as template.

The dsRNA from the green fluorescent protein encoded by *gfp* gene was obtained by performing colony PCR from *Escherichia coli* transformed with pGFPH (Ah-Fong & Judelson 2011). Positive colonies were grown overnight in 5 ml LB medium supplemented with 100 mg L^−1^ ampicillin. Plasmid isolation and purification was carried out using the Plasmid Midi Kit (Fermentas) following the manufacturer's protocol. A PCR with GFP-T7 primers was performed using the plasmids as template. Afterwards *gfp-*dsRNA and *SpTyr-*dsRNA were obtained using MEGAScript Kit (Ambion) according to manufacturer's protocol.

### dsRNA uptake in protoplasts

Three tubes were incubated at RT for 15 min: one tube with 10 μl Lipofectin (Invitrogen) and 10 μl *gfp-*dsRNA, a second tube with 10 μl Lipofectin and 10 μl *SpTyr-*dsRNA and a third tube with 10 μl Lipofectin and 10 μl of sterile RNase free water. Subsequently 10 μl of protoplasts solution containing 1 × 10^6^ protoplasts/ml was added to each tube and incubated at 18 °C for 16 h. Each experimental condition was then diluted in 200 ml Pea Broth and distributed in 2 ml aliquots in 24-well plates and incubated overnight at 24 °C.

The mycelium of regenerated protoplasts (of treated and non-treated with dsRNA) was then inoculated in PDA (potato dextrose agar, Oxoid) plates and incubated at 24 °C overnight before further processing.

### Real-time reverse-transcription quantitative PCR (qPCR)

For all dsRNA treated and control lines RNA extraction was performed as described in Section 2.3. Specific primers were designed for each gene using the Primer3 tool following the manufacturer's guidelines for primer design. The constitutively expressed gene Tubulin (*tub*) was used as an endogenous control in all qPCR reactions. A master mix was prepared containing per well: 25 μl LightCycler^®^ 480 SYBR Green I Master (Roche), 2 μl of each 10 mM primer [*tyr* 3′ACCTCTTCTACGGTCAGCA5’ and 3′AGGTTGTGCTAGTGGATCGG5’, *tub* 5′-AGGAGATGTTCAAGCGCGTC-3′ and 5′-GATCGTTCATGTTGGACTCGGC-3′(van West *et al.* 2010)] and 17 μl nuclease free water. Five μl of cDNA from each sample was added to the wells. Samples were run in triplicate. The standard error from all qPCR reactions was calculated for all individual lines tested and used to determine the confidence intervals. A melting curve was run for each primer set to confirm the reaction specificity.

### Tyrosinase activity assay

Sporulating mycelium from each individual line was ground with liquid nitrogen and added to 5 ml phosphate buffer 100 mM pH 6.5 containing: 500 μl PMSF 1 mM, 850 μl sorbitol 0.65 M, protease inhibitor EDTA free (Roche). Samples were centrifuged (10 min, 2100 g, 4 °C) and the supernatant (crude extract) collected into 15 mL tubes.

In new 1.5 ml tubes, 500 μl acetate buffer 50 mM pH 5.0, 200 μl l-DOPA 3.2 mM, 20 μl DMF 2 % (w/v) and 220 μl MBTH 5 mM were mixed and incubated 5 min at 37 °C. Thereafter 100 μl of crude extract was added and incubated for a further 30 min at 37 °C. Absorbance was measured at 505 nm and the activity determined using the equation below (Winder 1994): Rate (nmol min^−1^) = Δ*A505* *t*^−1^ × 10^3^/29 × 10^3^/30, where *t* = time.

### Melanin content determination

Sporulating mycelium was disrupted as described for the Tyrosinase assay. Samples were centrifuged 10 min at 2100 g and 4 °C and supernatant transferred to a new tube.

The absorbance of 1 ml of solution was measured at 475 nm. A standard curve was made using concentrations of commercial melanin (Sigma) ranging between 0 and 2 g l^−1^.

### Transmission electron microscopy (TEM)

Sporulating mycelium of control and putatively silenced lines were fixed in 3 % glutaraldehyde in 0.1 M phosphate buffer (PB) pH 7.4 for 24 h. Afterwards samples were washed and stored in 0.1 M PB pH 7.4 until further processing. For transmission electron microscopy (TEM) analysis, samples were subsequently processed in an automated routine tissue processor Leica EM TP (Leica Microsystems, Vienna, Austria) comprising following steps. Samples were post-fixed in 1 % (v/v) osmium tetroxide (OsO_4_; aqueous solution; code O004; TAAB, England, UK; Batch nr 70150) for 1 h preceded by three 5-min washes with PB and three 5-min washes with distilled water and an extra wash step for 30 min with distilled water. Next, samples were dehydrated in increasing concentrations of ethanol (30 %, 70 % and 95 %, and 100 % (v/v); 30 min each), followed by three incubations in acetone for 1 h. Samples were then incubated in increasing concentrations of epoxy/acetone (1/1, 6/1, and 100 % epoxy) for 1, 6 and 24 h respectively before embedding the samples in labelled capsules with freshly prepared resin, leaving the resin to polymerise for 24 h at 60 °C. Ultra-thin sections (70–80 nm) were cut with a Leica EM UC6 ultramicrotome (Leica Microsystems, Vienna, Austria) and mounted on 200-mesh uncoated copper grids. Grids were stained with 0.5 % uranyl acetate (Ultrastain1, ref. 705631095, Laurylab, France) and 0.5 % lead citrate (Ultrastain2, ref. 70553022, Laurylab, France) on an automated contrasting instrument Leica EM AC20 (Leica Microsystems, Vienna, Austria). Finally the grids were analysed at 80 kV using a JEM-1400 Plus (JEOL, Tokyo, Japan) transmission electron microscope equipped with an AMT UltraVUE camera.

## Results and discussion

Bioinformatic analysis of the *Saprolegnia parasitica* genome, (Broad Institute website), uncovered a gene that encodes a putative tyrosinase (Jiang *et al.* 2013) that, according to RNA sequence data, is highly expressed in sporulating mycelium. To verify this, quantitative expression analysis by real-time quantitative PCR of the different life stages was carried out demonstrating that the expression of *SpTyr* gene is ∼30 fold higher in sporulating mycelium compared to other *S*. *parasitica* life stages, confirming the initial RNA sequence data (de Bruijn *et al.* 2012).

**Fig 2:**
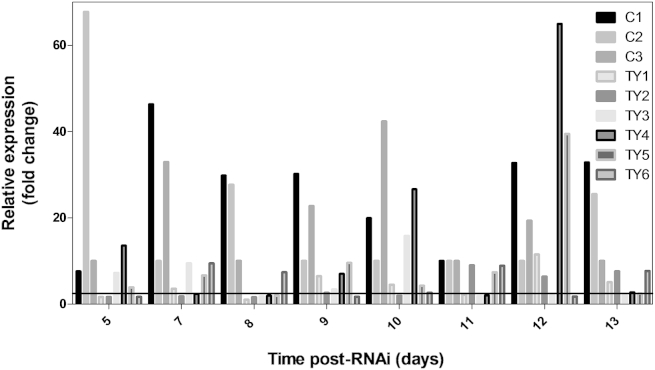
**The effect of *SpTyr-*dsRNA on Tyrosinase gene expression over time.** mRNA from sporulating mycelium of *Saprolegnia parasitica* was extracted after treatment with *SpTyr-*dsRNA and *gfp*-dsRNA over different time points as described in Material and Methods section. Gene expression levels of control lines, C, treated with *gfp*-dsRNA, and putative tyrosinase silenced lines, Ty, were assessed over 12 d by qPCR analysis and corrected for exogenously added mRNA of b-tubulin (housekeeping gene). This experiment shows a logarithmic tendency of gene expression on the putative silenced lines, where day 8 post-RNAi shows the highest silencing level for most of the putative *SpTyr*-silenced lines. Gene expression levels of the majority of the silenced lines reached a value compared to gene expression levels of the control by day 12.

To infer the function of a gene, several approaches can be used. One of the most efficient ones is gene silencing, where gene expression can be completely or partially abolished. Since the tyrosinase enzyme was expected to be involved in pigment formation, a decrease in gene expression would cause a visible change in the phenotype of the dsRNA treated individual lines. For transient gene silencing we used RNAi protocols that had already been established for *Phytophthora infestans* (Judelson et al., 1991, Whisson et al., 2005) and optimised it for silencing in *S*. *parasitica*. In order to maximize the change in gene expression of a silenced gene it is necessary to know the optimum time of silencing. The time line for this phenomenon differs according to the organism of study. To unravel the specific silencing timeline for *S*. *parasitica*, a time course RNAi assay was conducted for 12 d (Fig 2) and gene expression levels assessed by qPCR. The results of this experiment suggested that at 8 d after introduction of dsRNA into the cells, gene expression of *SpTyr* is down regulated the most in the majority of the individual lines tested. Five out of six individual lines (Ty1 – Ty5), treated with *SpTyr-*dsRNA (Fig 2) had less than 20 % gene expression when compared with the control lines. Individual line Ty6 was silenced within the time frame; however, the most significant decrease in gene expression occurred on day 9 post-RNAi.

These results suggest that the window of opportunity for gene silencing in *S*. *parasitica* CBS 223.65 is about 8–9 d after dsRNA uptake, which is much earlier than has been found for *P*. *infestans* where the maximum silencing was observed between 12 and 15 d after dsRNA uptake (Whisson *et al.* 2005). Each individual line obtained using RNAi technique is unique. With such a short timeframe and depending on the life stage where the gene of interest is most expressed, the amount of material from each individual line for further analysis is reduced.

**Fig 3:**
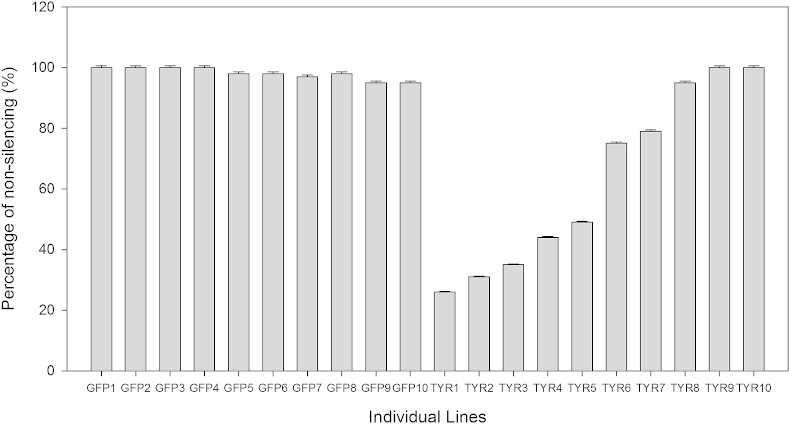
**Percentage of non-silencing obtained on individual lines using RNAi.** mRNA from lines *SpTyr-*dsRNA treated and *gfp*-dsRNA was extracted as described in Material and Methods. Gene expression levels of individual lines, after RNAi assay, were determined by qPCR analysis and corrected for exogenously added mRNA of b-tubulin (housekeeping gene). Its value is expressed in percentage of non-silencing. The percentage was calculated considering the control, *gfp-*dsRNA treated lines, as 100 % non-silenced. Note that *SpTyr-*dsRNA individual lines Tyr1 to Tyr5 present the higher level of silencing ranging between 60 % and 38 %. The error bars represent the standard error within three technical replicates.

After establishing the optimum time period for gene silencing in *S. parasitica* four RNAi assays were performed in order to optimise and increase the reproducibility of the technique. A final, fifth, optimised RNAi was conducted and ten individual lines treated with *gfp-*dsRNA (control) and ten individual lines treated with *SpTyr-*dsRNA were analysed by qPCR on the eighth day after dsRNA uptake to determine the level of silencing. Out of the ten individual lines treated with *SpTyr-*dsRNA, seven were silenced with gene expression levels reduced between 25 and 80 % of the levels of individual lines treated with *gfp-*dsRNA (Fig 3).

**Fig 4:**
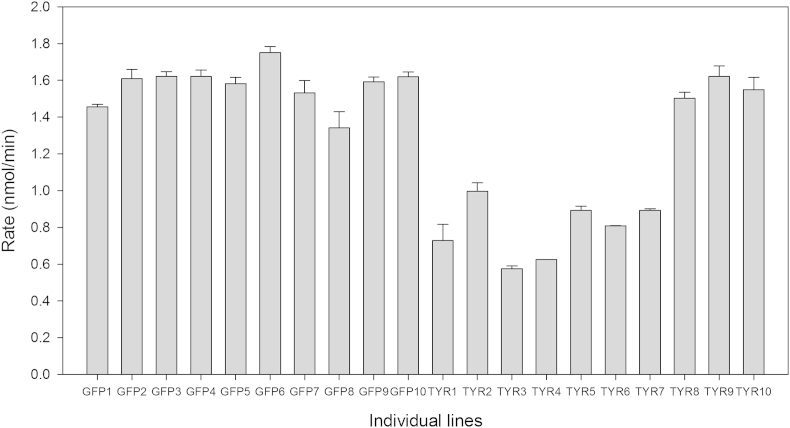
**Measurement of tyrosinase activity in individual lines from *Saprolegnia parasitica* treated with dsRNA.** Sporulating mycelium from all individual lines treated with dsRNA was collected and enzyme activity tested using a spectrophotometric assay (Winder 1994) as described in Material and Methods. The rate of oxidation of l-DOPA into dopaquinone by the tyrosinase was determined after 30 min of incubation at 37 °C. The amount of forming product is determined by measuring absorbance at 505 nm. *SpTyr-*dsRNA individual lines Tyr1 to Tyr7 present the lowest tyrosinase activity, corresponding to the highest silencing level achieved. Note that the same individual lines used to assess the gene expression level (Fig 2) were used for the enzyme activity measurements and melanin content analysis (Fig 5). The error bars represent the standard error within technical triplicates.

**Fig 5:**
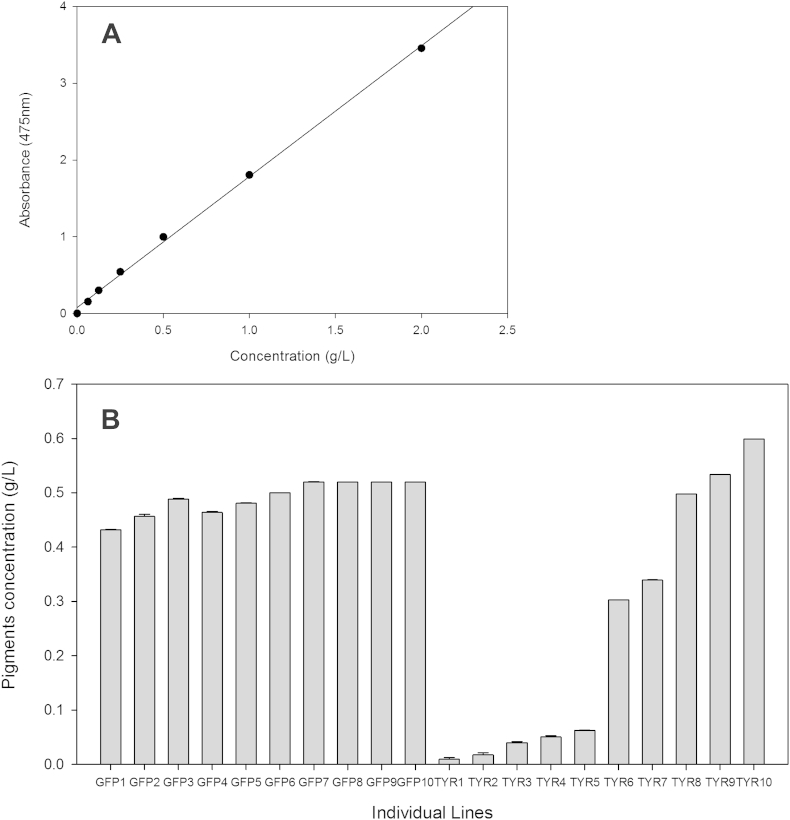
**Melanin content of individual lines from *Saprolegnia parasitica* treated with dsRNA.** Sporulating mycelium from all individual lines treated with dsRNA was collected and melanin content was determined using a spectrophotometric assay as described in Material and Methods section. The concentration was determined extrapolating the value through a standard curve from known concentrations of synthetic melanin. (A) Standard curve (B) melanin extrapolated concentration on individual lines treated with *gfp-*dsRNA and *SpTyr*-dsRNA. *SpTyr-*dsRNA individual lines Tyr1 to Tyr5 showed the lowest tyrosinase activity, corresponding to the highest silencing level achieved and to the lowest tyrosinase activity. Note that the same individual lines used to assess the gene expression level (Fig 3) and enzyme activity (Fig 4) were used for melanin content determination. The error bars represent the standard error within technical triplicates.

The same individual lines used for gene expression analysis were also tested for tyrosinase activity using a spectrophotometric assay. Tyrosinase activity in the individual lines that showed most silencing was decreased when compared to the control (Fig 4), while individual lines that did not present *SpTyr*-silencing or were at a low silencing level maintained a tyrosinase activity that was similar to the control lines. The lowest levels of tyrosinase activity were measured in the lines with the highest percentage of gene silencing. Additionally, melanin content of each individual line was determined spectrophotometrically and the concentration extrapolated from a standard curve (Fig 5A). The most silenced lines (lines Ty1 to Ty5) possessed less melanin when compared with the individual lines treated with *gfp-*dsRNA (Fig 5B). These results demonstrate that silencing the *SpTyr* gene affects the melanin production and demonstrate that the pigments present in *S*. *parasitica* contain melanin.

**Fig 6:**
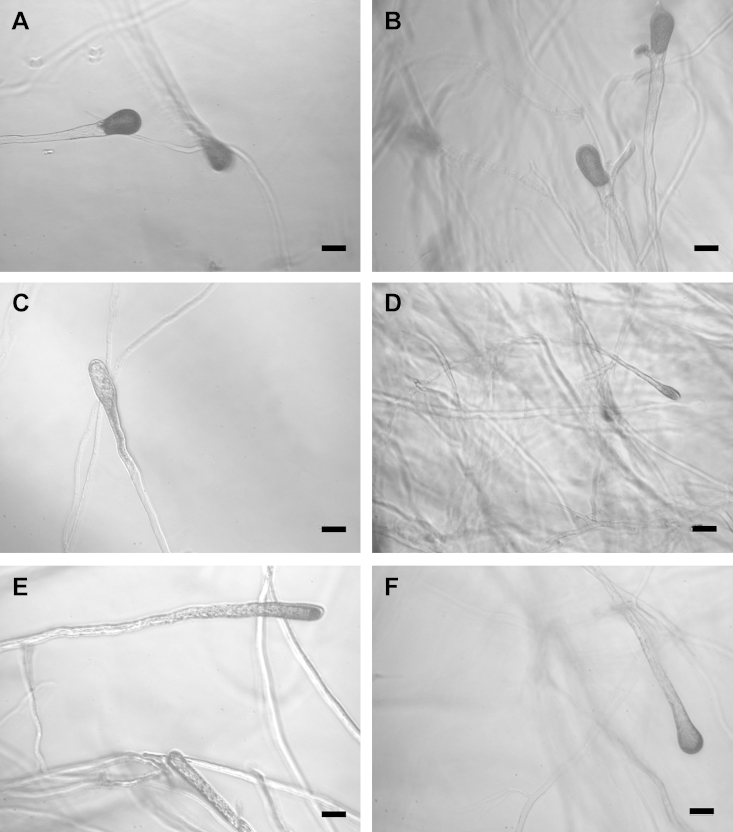
***Saprolegnia parasitica* sporangia phenotypes from different individual lines treated with dsRNA.** Phenotypes of individual lines of *Saprolegnia parasitica* treated with *SpTyr-*dsRNA were observed at 8 d after dsRNA-treatment. Individual silenced lines showed different phenotypes when compared with the control lines. Panels A and B (control lines) represent the normal morphology and colouration of young sporangia of *S. parasitica*. Panels C–F show some of the phenotypes observed in different *SpTyr-*dsRNA-treated lines, whereby some young sporangia have less pigmentation (C), or are smaller (D), or are elongated with less pigments (E) or sporangia that are pigmented only at the tip (F). Scale bar represents 100 μm.

Furthermore, microscopic analysis revealed some altered sporangium morphology in the most-silenced lines when compared with *gfp*-silenced lines and the wild type strain. *SpTyr*-silenced lines seem to have less pigmentation, smaller sporangia and also abnormally shaped sporangia (Fig 6). We observed these differences in both young and mature sporangia (data not shown). The effect of tyrosinase silencing on sporangial morphology may be due to the pigments being deposited in or close to the cell wall, helping the structure thickness and shape. However, we cannot infer that this altered morphology is solely due to the silencing of *SpTyr* gene. For example it is possible that a pleiotropic effect, resulting in altered sporangial morphology, was obtained due to *SpTyr*-silencing. This has been described before when an RNA-helicase of *P*. *infestans* was silenced with RNAi (Walker *et al.* 2008; Walker and van West, 2007).

**Fig 7:**
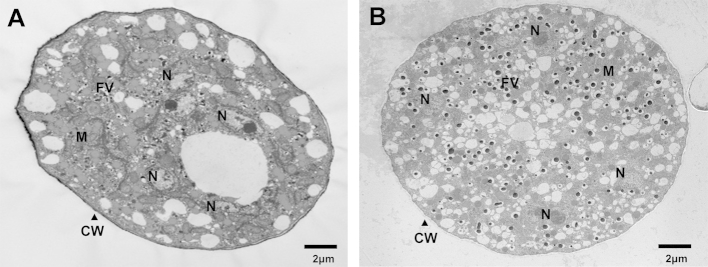
**Transmission electron microscopy studies of dsRNA treated lines derived from wild-type strain CBS223.65.** Panel A: A young sporangium of a *gfp*-dsRNA-treated line containing several nuclei (N) and fingerprint vesicles (FV), Scale bar represents 2 μm. Panel B: A young sporangium of a *SpTyr*-dsRNA-treated line containing several nuclei (N), mitochondria (M) and fingerprint vesicles (FV) Scale bar represents 2 μm.

**Fig 8:**
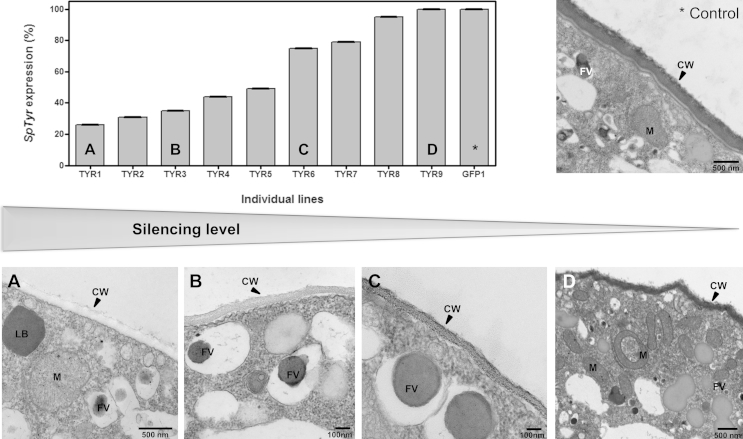
**Effect of *SpTyr*-silencing on *Saprolegnia parasitica* cell wall.** The gene expression level for all individual lines was obtained by qPCR analysis using exogenous β-tubulin as housekeeping gene. The level of silencing achieved by the putatively silenced lines was transformed into percentage of remaining *SpTyr*-expression after silencing (in graph referred to as ‘*SpTyr*-expression’) having the minimal gene expression level obtained for the control lines (*GFP-dsRNA*) as 100 % non-silenced (upper left graph). A range of putatively *SpTyr*-silenced lines (panel A to D) as well as control lines (panel *) were prepared and analysed using TEM. This procedure revealed an electron dense layer in the cell wall (CW) of sporangia of the control lines (*) and a non-silenced line (D). The electron dense layer decreased in the cell wall of the sporangium with decreased levels of *SpTyr*-expression (images A–C).

Transmission electron microscopy (TEM) was used to study the effect of *SpTyr*-silencing on sporangium morphology in more detail. Young sporangia were analysed from both *gfp-*dsRNA-treated lines (control) and *SpTyr-*silenced lines after induction of sporulation. No significant differences in the cellular structures and organelles of control and *SpTyr*-silenced sporangia were observed. In both control and *SpTyr*-silenced lines, sporangia were multinucleated and fingerprint vesicles can be observed (Fig 7). The sporangial cell wall of the control lines and the weakly *SpTyr*-silenced lines are more electron dense than the most *SpTyr*-silenced lines (Fig 8). These observations would suggest that melanin is located within the cell wall, which has been demonstrated in some true fungi (Wang et al., 1995, Casadevall et al., 2000, Eisenman et al., 2005). However it is still possible that other organelles might also contain melanin.

Abnormally shaped sporangia were also observed in *SpTyr*-silenced lines using TEM, whereby elongated and even triangular shaped sporangia were formed (data not shown). In both types of sporangia an increase in vacuoles and cytoplasm was found when compared to control lines. The increase in vacuoles could also explain the altered morphology of the sporangia observed with the light microscope.

## Conclusions

The tyrosinase gene, *SpTyr*, of *Saprolegnia parasitica* is integral to the melanin biosynthetic pathway of this oomycete. After silencing the tyrosinase gene, melanin production was reduced because of a decrease in tyrosinase activity. Microscopical analysis suggest that melanin is located in an electron-dense layer in the cell wall of sporangia of *S*. *parasitica*, since the electron dense layer was absent in the most silenced lines. With this work, we have demonstrated for the first time that transient gene silencing through RNAi is a feasible method to functionally characterise genes in *S*. *parasitica*.
